# Patient-provider communication about pregnancy and HIV among female sex workers living with HIV in Santo Domingo, Dominican Republic

**DOI:** 10.1186/s12884-019-2482-5

**Published:** 2019-11-21

**Authors:** Dana Cernigliaro, Clare Barrington, Yeycy Donastorg, Martha Perez, Deanna Kerrigan

**Affiliations:** 1 The Johns Hopkins Bloomberg School of Public Health, 624 N Broadway HH 257, Baltimore, MD 21205 USA; 2 The University of North Carolina Gillings School of Global Public Health, Rosenau Hall 319B, CB#7440, Chapel Hill, NC 27599 USA; 3 Instituto Dermatologico y Cirugia de la Piel, Albert Thomas, 66, Santo Domingo, Dominican Republic; 4 Center on Health, Risk & Society, American University, 4400 Massachusetts Avenue, Washington, DC, NW 20016 USA

**Keywords:** Sex workers, HIV, Pregnancy, Reproductive health, Patient-provider communication, Dominican Republic

## Abstract

**Background:**

Health providers can play an important role in communication about pregnancy, particularly for women at increased risk for pregnancy complications, including female sex workers (FSWs) living with HIV. This study explored factors related to patient-provider communication about pregnancy among 253 FSWs living with HIV of reproductive age in Santo Domingo, Dominican Republic.

**Methods:**

A cross-sectional design was employed including structured socio-behavioral surveys. Data were analyzed utilizing bivariate and multivariate logistic regression.

**Results:**

Of the 253 FSWs living with HIV in this study, 95.7% had been pregnant at least once (median: 4; IQR: 3,6), 28.0% wanted more children and 36% reported a pregnancy after HIV diagnosis. Over half of participants (58.0%) reported having ever spoken to a health provider about pregnancy while living with HIV. Multivariate logistic regression found significant associations between having spoken to a health provider about HIV in pregnancy and a more positive perception of their provider (AOR: 2.0; 95% CI: 1.0, 2.5) and years since HIV diagnosis (AOR: 1.1; 95% CI: 1.0, 1.1). Participants were less likely to speak with a provider if they had a history of drug use (AOR: 0.4; 95% CI: 0.2, 0.9) or current alcohol use (AOR: 0.5; 95% CI: 0.3, 0.9).

**Conclusion:**

Findings highlight the importance of non-judgmental and tailored provider-initiated conversations surrounding pregnancy. Future research is needed to better understand how and when pregnancy communication is initiated, as well as the content of clinical care conversations, to address the reproductive health of FSWs living with HIV.

## Background

Pregnancy rates among women living with HIV have significantly increased over the last 10 years across geographic settings, giving increased salience to preconception conversations surrounding pregnancy [1, 2]. Conversations surrounding pregnancy care and counseling by a health provider can address the increased risks that women living with HIV and their infants face, therefore leading to improved health outcomes [3–5]. For women living with HIV who desire more children, preconception care and counseling provides an opportunity to promote planned pregnancies, dispel fears about pregnancy and HIV, educate women on prevention of mother to child transmission of HIV (MTCT) through ART adherence and retention in care, and help ensure safe outcomes for both mother and child [5–8]. Further, for women who do not want to get pregnant, discussing pregnancy and family planning can prevent unwanted pregnancies and potential negative mental and physical health outcomes.

When conversations surrounding pregnancy do occur between women living with HIV and their health providers, they are frequently patient-initiated and occur after pregnancy [6, 8, 9]. Health care workers’ methods of communication and their attitudes have been seen to influence proactive communication and personal decisions surrounding pregnancy and fertility among women living with HIV [10–12]. If women living with HIV perceive negative attitudes, stigma or discrimination about childbearing from their health provider, they are more likely to have negative attitudes themselves and may avoid discussing fertility [12, 13]. In many settings, however, a majority of HIV-infected women of reproductive age have never discussed pregnancy and childbearing with their health providers, highlighting a clear gap in service provision [6–8].

Reasons for this gap and for the lack of pregnancy communication between health providers and women living with HIV can be seen from both the provider and patient perspective. From a patient perspective, women may be more concerned with competing priorities and more immediate concerns when seeing a health provider. Women may also feel stigmatized or judged due to their HIV infection or lack a sense of empowerment to discuss sexual health [13]. Even if women living with HIV feel that they can conceive with appropriate care, they may still feel societal stigma at having children, adding to difficulty in initiating the conversation [8]. Providers may be reluctant to discuss reproduction and fertility or may assume that women living with HIV do not want to become pregnant [13]. From a dual perspective, there may be lack of knowledge about pregnancy in HIV by either patient or provider, or a lack of defined provider roles if the patient is seeing multiple providers for care [13].

In addition to preconception communication surrounding pregnancy, challenges in communication have been seen to continue through care and treatment after pregnancy [14]. For example, patient-provider interactions during pregnancy related to ART initiation and adherence [12] have been found to include negative factors such as stigma and discrimination, a biomedically-focused conversation without addressing social factors [15] and power dynamics between patient-provider that affect a woman’s ability to ask questions [16].

Within the body of research focused on patient-provider communication for women living with HIV, there has been limited attention to marginalized populations, such as female sex workers (FSWs). Globally, many FSWs have children and have been pregnant [17, 18]. Similar to women living with HIV, FSWs face severe discrimination from health care workers and other barriers to care in different geographic settings [19–22]. At the same time, FSWs are at greater risk for a number of health concerns including HIV, other STIs, gender-based violence [18, 23, 24] and complications associated with reproductive health, pregnancy and childbearing [25, 26].

The global burden HIV for FSWs is significant, with FSWs having approximately 13.5 greater odds of being HIV-infected than women in the general population in lower and middle-income countries [27]. Additionally, FSWs living with HIV are faced with multiple stigmatized identities. They are more likely to experience discrimination in health care settings, report feelings of social isolation once they learned their HIV sero-status, face refusal of medical care or fear of seeking services compared to FSWs not living with HIV [21]. Prior research has found that FSWs who desire children are more likely to have higher levels of HIV-related internalized stigma, a history of pregnancy loss and perceive that their partner has negative feeling about a pregnancy [28]. FSWs who become pregnant after HIV diagnosis are more likely to have ART interruption and have a more negative perception of their health provider [29]. Acknowledgement of the need for comprehensive and tailored prevention, care and treatment for this population is understood by the global community [22]. This paper aims to understand factors related to patient-provider communication about pregnancy among FSWs living with HIV in Santo Domingo, Dominican Republic (DR).

## Methods

### Study context

The current analysis is from baseline data of a longitudinal multi-level intervention study named *Abriendo Puertas* (Opening Doors). The aim of *Abriendo Puertas* was to assess the feasibility and initial effects of a multi-level intervention to promote engagement in and adherence to HIV care and foster preventive sexual health behaviors for FSW living with HIV through counseling, education, peer navigation, provider engagement and community mobilization [30]. Sex work is not illegal in the DR and organizations exist whose aim is to educate and empower FSWs. FSWs in the DR are disproportionately affected by HIV, with a prevalence estimated at 4.8%, [23] as compared to 0.7% for the country [31].

### Study sample, collaborative partners and recruitment

This study collaborated with partners in Santo Domingo to conduct formative research, including Movimiento de Mujeres Unidas, a sex worker rights group and Centro de Orientacion e Investigacion Integral, an HIV prevention organization and a local research unit. Study participants were recruited through convenience sampling techniques. Participants were identified through peer navigators at collaborating HIV clinics and through the navigator’s community outreach, as well as through referrals by other study FSWs living with HIV in the study. FSWs were defined as women who report having exchanged sex for money in the last month. Eligibility criteria included being at least 18 years of age, spoke Spanish and reported HIV-infection, confirmed prior to enrollment by a HIV rapid test. Enrollment occurred from November 2012 to February 2013, resulting in a sample size of 268 FSWs living with HIV. Details of recruitment and study design have been described previously [28–30]. This analysis includes a subset of FSWs who were of reproductive age while living with HIV for a total of 253 FSWs living with HIV.

### Data collection

This baseline socio-behavioral survey was conducted by trained Dominican field staff in Spanish within private offices of the HIV Vaccine Research Unit (HVRU) of the Instituto Dermatologico y Cirugia de la Piel Dr. Humberto Bogart Diaz (IDCP). Blood samples for HIV viral load were assessed at the Dominican National Reference Laboratory in Santo Domingo using polymerase chain reaction (PCR) testing.

### Ethical consideration

Oral consent was provided and participants were compensated 400 Dominican pesos (about $10) for survey completion. Interviewers signed IRB approved consent forms indicating participant’s verbal consent to both the survey and blood draw for the study. Approval was granted by the following Institutional Review Boards: Johns Hopkins Bloomberg School of Public Health, University of North Carolina and the Instituto Dermatologico y Cirugia de la Piel Dr. Humberto Bogart Diaz.

### Measures

#### Dependent variable

The main outcome was assessed through the following question, “Have you and a health care provider ever talked about pregnancy in women living with HIV?” Response options were “yes” “no” “don’t know” and “refuse to answer”. All respondents answered either yes or no and therefore treated as a dichotomous variable.

Key independent variables: Selection of independent variables was guided though background literature and conceptual relevance. The main independent variable was perception of your main health provider, a scale from 1 to 4 that included eight questions, such as “You feel respected by your doctor” and “Your feel comfortable asking your doctor questions about your care”. A higher score meant greater satisfaction with your health provider. Other independent variables included socio-demographic characteristics (age, civil status, education), risk behaviors (alcohol use, drug use ever), HIV characteristics (years since diagnosis, viral load), stigma scales (HIV related internalized stigma, sex work related internalized stigma) and sexual and reproductive health characteristics (number of pregnancies, contraception use, pregnant since diagnosis, attitudes about pregnancy and HIV, knowledge or MTCT, and a desire for more children).

#### Aggregate measures

Consistent condom use was an aggregate of three separate questions asking FSWs living with HIV if they: 1) ever had sex without a condom 2) had sex without a condom in the last 30 days and 3) had sex without a condom the last time they had sex. These questions were asked for 3 types of partners (regular partner, casual partner, client). If FSWs living with HIV answered that condoms were consistently used for each partner this was categorized as consistent condom use. Stigma scales included sex work and HIV-internalized stigma scales. Scales were developed using adapted measures from Berger et al., [32] Zelaya et al., [33, 34] and Baral et al., [35] and with guidance from Earnshaw’s HIV Stigma Framework [36]. All stigma scales included options of 1 to 4, with1 being the least amount of stigma and 4 the most stigma. Statements such as “Having HIV makes you feel like a bad person” or “You feel ashamed that you have HIV” among others were included in the HIV stigma scale, while the sex work stigma scale asked similar questions about sex work. Response options included: 1 = totally disagree, 2 = disagree, 3 = agree, 4 = totally agree, 88 = don’t know and 99 = refuse to answer. All answers of “don’t know” and “refuse to answer” responses were coded as 2.5 to maintain sample size but neutralize their weight. Standardization of directionality was ensured for all questions. The provider satisfaction scale, the primary independent variable, was adapted from the validated Patients Reactions Assessment scale [37] differed in that a higher score meant a greater satisfaction with their providers. Data reduction occurred through principal components analysis. Eigenvalues of ≥1 were considered, along with scree plots and parallel factor analysis results, followed by a test of normality. Factors were rotated and if factor loadings were less than 0.4 and uniqueness was greater than 0.5, the factors were dropped. Once items were chosen for removal, Cronbach alpha tests were conducted (see Table 1) in order to measure internal consistency of the final scale. When scales were finalized, the items included were averaged across participant to create each final variable.

**Table 1:** Stigma and provider scale characteristics

Scale name	Number of final items	Cronbach alpha score
HIV related self-internalized stigma scale	7	0.88
Sex work related self/ internalized stigma scale	12	0.91
Provider satisfaction scale	8	0.96

### Data analysis

Data was collected and uploaded into a secure SQL server database and converted to Stata version 11 for analysis. Exploratory analysis was conducted and any outliers or inconsistencies were checked against original data. T-tests or chi-square analysis, as appropriate, were used to explore associations between independent variables and the outcome. Bivariate logistic regression was conducted to determine odds ratios and confidence intervals for each independent variable. Associations that were determined at a 0.10 *p*-value level or less and guided by background literature and conceptual relevance were included in the model. The final model was built using multiple iterations through a traditional stepwise approach. After the addition of each variable the Akaike information criterion (AIC) was calculated for each nested model and log likelihood tests were assessed. The model with the most parsimonious fit- the lowest AIC value, together with low log likelihood values aided in the determination of the final model. Finally, a Hosmer-Lemeshow goodness of fit test was then conducted to determine the fit of the final model.

## Results

### Descriptive characteristics

The final sample size was 253 female sex workers living with HIV, with a median age of 35 years (interquartile range (IQR): 30, 40). Table 2 illustrates socio-demographic, behavioral, sexual health, HIV and stigma characteristics. Almost all women had some education (98.4%), most at the primary level (61.3%) and most were married or had a partner (81.8%). The median age reported for first engaging in sex work was 18 years (IQR: 15, 23) with a majority reporting they were under 20 years of age. Predominant sex work locations included establishment-based (e.g. disco, bar, hotel, billiard) or the street, however many indicated more than one location. A majority (59.7%) reported never or rarely drinking alcohol. More than half (63.6%) reported consistent condom use with all sexual partners. Number of years since HIV diagnosis was broad (mean: 6.0; range: < 1 year, 30 years), however, 63.2% were diagnosed in the last 6 years.

**Table 2:** Socio-demographic and health characteristics of FSWs living with HIV (*n* = 253)

Socio-demographic characteristics	N	Percent	Median (IQR)	Mean ± SD
Age			35 (30, 40)	
Civil status				
Single/widowed or divorced no regular partner	46	18.2		
Married or have a partner	207	81.8		
Education (any)	249	98.4		
No education	4	1.6		
Primary education	155	61.3		
Secondary or tertiary	94	32.2		
Current Residence				
Santo Domingo	196	77.5		
Other area	57	22.5		
Sex work and risk behavior characteristics				
Age first engaged in sex work			18 (15, 23)	
Average price per salida (pesos)				886 ± 534^a^
Number of clients/wk.			3 (2, 6)	
Work Locations				
Establishment	155	59.7		
The street	140	55.3		
Other (predominantly by calling)	80	31.6		
Any conflict with a sex partner in the last 6 months	92	36.4		
Alcohol use				
Sometimes/often	102	40.3		
Rarely/never	151	59.7		
Drug use (ever)	62	24.5		
Sexual health and HIV care				
Consistent condom use with all partners (*n* = 250)	159	63.6		
Permanent contraceptive procedure after diagnosis	74	29.4		
Viral Load (*n* = 249) Undetectable (< 50 copies/cc)	119	47.8		
Detectable	130	52.2		
Current ARV use (*n* = 252)	182	72.2		
Years since HIV diagnosis				6.0 ± 4.7
Stigma and provider scales (*n* = 253) ^b^				
HIV related self-internalized stigma scale (20)				2.4 ± 0.6
Sex work related self-internalized stigma scale (19)				2.4 ± 0.6
Perception of provider scale				3.2 ± 0.6

^a^ 870 pesos ~ $20 ^b^Stigma scales: 1 is the lowest amount of stigma and 4 the highest. Provider scale: a higher scale indicates better perception of provider

### Reproductive health and pregnancy

Reproductive health and service characteristics are illustrated in Table 3. Almost all of the women were pregnant at least once (95.7%) and had children (93.3%) with a median of 3 children (IQR: 2, 4). Many women reported at least one pregnancy since diagnosis (36.0%), with a median of 4 pregnancies (IQR: 3,6). About 28.0% indicated a desire for (more) children. A high number of women (158/241) 65.6%) reported a pregnancy that did not result in a live birth. Over half did not feel that it is good to get pregnant if you are living with HIV and desire more children (56.1%). Barriers to care included feelings that lack of access to treatment and HIV care was a serious problem for FSWs (75.0%), having ever had a fear of seeking health services due to HIV or sex work (44.4%), confusion with provider communication about treatment (31.9%) and fear that their provider may not keep their information private (30.0%). Communication with a health provider occurred for more than half (57.9%) and clear gaps in communication were seen for women in most need of this discussion. For example, gaps in communication occurred among women who had previously spoken to a healthcare provider about pregnancy in HIV-positive women and had been pregnant since HIV diagnosis (56/146, 38.6%), wanted more children (36/146, 24.7%) and those who felt it was not good to get pregnant if you are living with HIV (83/146, 56.4%).

**Table 3:** Reproductive health and health service characteristics among FSWs living with HIV (*n* = 253)

Reproductive health and health service characteristics	N	Percent	Median (IQR)	Mean ± SD
Currently have children	236	93.3		
Number of children (*n* = 236)			3 (2, 4)	
Have ever been pregnant in lifetime	242	95.7		
Number of times pregnant (*n* = 242)			4 (3, 6)	
At least one pregnancy loss (*n* = 241)	158	65.6		
Have been pregnant since HIV diagnosis	91	36.0		
Number of times pregnant (*n* = 91)				1.6 ± 0.9
At least one pregnancy loss since diagnosis (*n* = 91)	33	27.7		
Desire to have more children (yes or maybe)	70	28.3		
Number of children desired (*n* = 68)				1.6 ± 0.9
Currently/possibly pregnant	8	3.2		
Do not feel that if an HIV positive woman wants to get pregnant she should	142	56.1		
Knows that ARTs can reduce the risk of MTCT	192	75.9		
Feels health providers are supportive of pregnancy in women living with HIV	191	75.5		
Services and health care				
Feel that a lack of access to treatment and HIV care is a serious problem for SW	185	75.2		
Have ever been afraid to seek health services due to HIV or sex work	112	44.4		
Worry that people will find out my HIV status if I attend the health clinic (*n* = 252)	81	32.1		
When your doctor gives information about treatment you come away feeling confused	73	31.9		
Feels that health providers might not keep information private	75	28.0		
Reproductive health access to care and services				
Have ever spoken to a healthcare provider about pregnancy in HIV positive women (*n* = 252)	146	57.9		
And have been pregnant since HIV diagnosis	56	38.6		
And would like/might like another child	36	24.7		
And who disagree that it is good to get pregnant if you are HIV positive	83	56.4		
Have been pregnant since diagnosis without discussing pregnancy (91–56 = 35; 35/91)	35	38.4		
Would like (more) children and have not discussed pregnancy (70–36 = 34; 34/70)	34	48.6		
Did not use any method to prevent pregnancy in the last 6 M (*n* = 252)	45	17.9		
And do not want more children	27	60.0		

### Associations with having spoken to a health provider about HIV and pregnancy

Bivariate and multivariate regression analysis results are highlighted in Table 4. Bivariate regression analysis revealed associations at the 0.05 level of significance or less between female sex workers who had spoken to a health provider about pregnancy among women living with HIV and those reporting substance use (ever having used drugs (OR: 0.3; 95% CI: 0.2, 0.6) or current often/occasional alcohol use (OR: 0.5; 95% CI: 0.3, 0.8)) and women with a better perception of their health provider (OR: 2.0; 95% CI: 1.3, 3.0). Both stigma scales had non-significant negative associations with the outcome, with association of HIV internalized stigma only marginally non-significant (OR:0.7; 95% CI: 0.4, 1.1). Controlling for age, civil status and education, years living with HIV and number of times pregnant, the multivariate analysis uncovered a number of associations with having ever spoken to a health care provider about HIV and pregnancy at the 0.05 level of significance. Female sex workers living with HIV who had a conversation about HIV and pregnancy with their health provider were more likely to have a more positive perception of their health providers (AOR: 1.6; 95% CI: 1.0, 2.5), less likely to have had alcohol use in the last 30 days (AOR: 0.5, 95% CI: 0.3, 0.9) and are less likely to have a history of drug use (AOR: 0.4, 95% CI: 0.2, 0.8).

**Table 4:** Bivariate and multivariate logistic regression analysis for having spoken to a health provider about pregnancy among FSWs living with HIV (*n* = 252)

Sociodemographics	OR	95% CI	AOR	95% CI
Age	1.0	1.0, 1.0	1.0	0.9, 1.0
Civil status (ref: has partner)	0.7	0.4, 1.3	0.9	0.4, 1.7
Education (ref: secondary/tertiary education)	1.6	0.9, 2.7	1.3	0.7, 2.3
Risk behaviors and HIV characteristics				
Alcohol use (last 30 days)	0.5**	0.3, 0.8	0.5*	0.3, 0.9
Drug use ever	0.3***	0.2, 0.6	0.4*	0.2, 0.8
Years since HIV diagnosis	1.1*	1.0, 1.1	1.1*	1.0, 1.1
Viral load (*n* = 248)	0.6	0.4, 1.0		
Sexual and reproductive health characteristics				
Number of times pregnant	0.9	0.8, 1.0	0.9	0.8, 1.1
No contraception in the last 6 months	0.8	0.4, 1.5		
Have been pregnant since HIV diagnosis	1.3	0.8, 2.1		
If an woman with HIV wants to get pregnant she should try	0.9	0.5, 1.5		
Knows that ART during pregnancy can prevent MTCT	1.1	0.6, 2.		
Desire more children	0.7	0.4, 1.2		
Stigma and support				
Perception of provider scale	2.0**	1.3, 3.0	1.6*	1.0, 2.5
HIV related internalized stigma scale	0.7	0.4, 1.1		
Sex work related internalized stigma scale	0.9	0.6, 1.4		

* *p* ≤ 0.05, ** *p* ≤ 0.01, ***p ≤ 0.001

## Discussion

This study aimed to understand factors related to communication with health providers among female sex workers living with HIV in the DR. Results highlight that while pregnancy and childbearing are prevalent in these women’s lives, there are clear barriers to provider communication about pregnancy, as has been documented previously in other studies of women living with HIV [6, 8]. Many FSWs living with HIV who have been pregnant since diagnosis and who want more children have never spoken to a provider about pregnancy. Pregnancy, pregnancy desire, and having children are prevalent among these women. However, this population is at high risk for pregnancy and postpartum related health issues and therefore is most in need for constructive, non-judgmental conversations with their health provider.

Substance use (both current alcohol use and history of drug use) was a significant deterrent to provider communication about pregnancy. Substance use has also been associated with ART interruption, STI diagnosis and having detectable viral load for women living with HIV [30, 38]. Since substance use is also a stigmatized behavior, a population that is dealing with multiple stigmatized identities may avoid discussing pregnancy for fear of judgment. It is important to consider how substance use may further inhibit self-initiated or provider-initiated communication about pregnancy based on explicit and implicit biases among female sex workers living with HIV who are also substance users.

We also found that female sex workers living with HIV who had a more positive perception of their providers were significantly more likely to have spoken to a health provider about pregnancy, possibly because they felt that they will not be judged by their provider about their reproductive health decisions. Provider verbal and non-verbal attitudes have been seen to be influential on the desire to discuss pregnancy and personal decision-making surrounding pregnancy among women living with HIV [10–12, 39]. For these women who have multiple reproductive health concerns and who face multiple barriers to care, the need for non-discriminatory care and counseling from healthcare and HIV clinical providers is critical for both maternal and child health.

Lack of patient-provider communication about pregnancy has been seen among women living with HIV in other settings [6–8, 40], however FSWs may be at greater risk pregnancy related complications [25, 26]. The lack of patient-provider communication surrounding pregnancy in this study is concerning due to the high reported number of lifetime pregnancies, number of children and pregnancies that did not result in live births. These women were all of reproductive age when diagnosed with HIV and had seen a health provider at least once, highlighting missed opportunities for education and tailored care. Many FSWs living with HIV reported not using any contraceptive methods to prevent pregnancy in the last 6 months and reported not wanting more children, highlighting an unmet need for conversations surrounding family planning. Additionally, studies have found that female sex workers living with HIV who want children are more likely to have HIV-related internalized stigma and a history of pregnancy loss, [28] while those who have been pregnant after HIV diagnosis are more likely to have a negative perception of their health provider and have ART interruption [29].

We did not find a significant association between HIV or sex work internalized stigma and speaking to a provider about pregnancy in this study. Many female sex workers may not disclose their sex work to providers for fear of stigma or judgment, [19–22] which may explain the lack of association with the sex work related internalized stigma scale. Other studies have also found influences between HIV related stigma and patient-provider communication [8, 13] and the desire for more children [29].

Patient-provider communication regarding reproductive health and pregnancy is essential on multiple levels- to reduce stigma, to tailor care, and to provide appropriate support for women living with HIV. While the majority of female sex workers living with HIV had spoken to a health care provider about pregnancy and HIV, it is of concern that numbers were lower among HIV-positive women who had been pregnant since HIV diagnosis, who wanted more children and who had negative perceptions of pregnancy and HIV. Further, the fact that most women felt that lack of access to treatment and care was a problem for sex workers and many have been afraid to seek services due to HIV or sex work- further research is needed to explore dynamics between female sex workers living with HIV and health providers.

Proper preconception conversations with providers can dispel anxiety, encourage adherence and ultimately improve maternal and child health as well as help avoid unwanted pregnancies and prevent unsafe abortion [5]. In a country that where there are strict legal barriers to safe abortion, [41] a history of high maternal and infant mortality rates [42] and a history of questionable maternity wards practices, [43] understanding barriers to patient-provider communication is even more salient for these women, who face multiple stigmatized identities.

There are several limitations that should be discussed with regard to this study. First this study is cross-sectional in nature and therefore only captures participant information at one point in time. There may be issues with self-report or recall bias for many questions and a level of social desirability bias for issues surrounding health care or behavior. Future studies should focus on nuances of patient-provider communications surrounding pregnancy among these women including whether conversations are patient or provider initiated or if these conversations occur pre or post conception. Qualitative studies would also be helpful to further understand the context and details of these conversations. With these limitations and gaps in mind, this study did highlight important factors related to patient-provider communication about pregnancy for a key population that is at high risk for unintended pregnancy, MTCT and health complications during pregnancy. Interventions with health providers are needed to ensure safe and respectful conversations surrounding family planning for a population that is in significant need of comprehensive reproductive health services.

## Conclusions

Female sex workers living with HIV face a number of challenges and barriers to care for reproductive health. FSWs with a history or current substance use may be less likely to have conversations about HIV and pregnancy with their provider, but they are at greater risk for pregnancy complications. Non-judgmental provider-initiated conversations surrounding pregnancy and family planning are essential for a safe pregnancy outcomes. Health providers sensitized to these concerns can play a critical role in promoting the health and well-being of FSWs living with HIV and their families. Tailored services and interventions that address multiple levels of risk for FSWs living with HIV are crucial, as this population faces unique health concerns and barriers to care.

## Data Availability

Data available upon request from Dr. Deanna Kerrigan, American University, 4400 Massachusetts Avenue, Washington DC NW 20016. USA. Email: kerrigan@american.edu.

## Abbreviations

COIN: Centro de Orientacion e Investigacion Integral
DR: Dominican Republic
IDCP: Instituto Dermatologico y Cirugia de Piel Dr. Humberto Bogart Diaz
MODEMU: Movimiento de Mujeres Unidas
PCR: Polymerase chain reaction
